# Adaptation of Eurasian Magpie (*Pica pica*) to Urban Environments: Population Dynamics and Habitat Preferences in Zielona Góra (Poland) over 23 Years

**DOI:** 10.3390/ani15050704

**Published:** 2025-02-28

**Authors:** Olaf Ciebiera, Paweł Czechowski, Federico Morelli, Sławomir Rubacha, Leszek Jerzak

**Affiliations:** 1 Institute of Biological Sciences, University of Zielona Góra, 65-516 Zielona Góra, Poland; 2 Institute of Sport, Tourism and Nutrition, University of Zielona Góra, 65-516 Zielona Góra, Poland; 3 Owls Conservation Association, 65-119 Zielona Góra, Poland

**Keywords:** *Pica pica*, magpie, abundance, density, habitat selectivity, Poland

## Abstract

This study investigates the changes in population size, distribution, and habitat preferences of the Eurasian magpie *Pica pica* in Zielona Góra over 23 years, emphasising the effects of urbanisation and habitat transformation. A comprehensive survey conducted in 2022 identified 953 magpie pairs, with an average density of 8.8 pairs/km^2^ across the current administrative boundaries of Zielona Góra (without forests), and 27.7 pairs/km^2^ in strictly urbanised zones. The highest densities were observed in the old town (36.5 pairs/km^2^) and residential blocks (34.5 pairs/km^2^), while peripheral areas, like allotment gardens and industrial zones, showed significantly lower densities. The nests were predominantly located in coniferous trees, especially spruces, marking a shift from the previously favoured poplars. The mean nest height was 11.8 m, varying by habitat type, with the highest nests found in the old town and parks. Environmental factors, such as proximity to trash bins, water sources, and tall trees, were significant predictors of nest density and placement. These findings underscore the magpie’s adaptability to urban environments, influenced by the availability of anthropogenic resources, habitat structure, and surrounding urban features.

## Simple Summary

This study examines the population dynamics and habitat preferences of the Eurasian magpie *Pica pica* in Zielona Góra over 23 years, highlighting the impact of urbanisation. In 2022, 953 magpie pairs were recorded, with an average density of 8.8 pairs/km^2^ across the current administrative boundaries of Zielona Góra (without forests), and 27.7 pairs/km^2^ in strictly urbanised zones. The highest densities were in the old town (36.5 pairs/km^2^) and residential blocks (34.5 pairs/km^2^), while the peripheral areas had lower densities. The nests were mostly in coniferous trees, especially spruces, indicating a shift from poplars. The average nest height was 11.8 m, varying by habitat, with taller nests in the old town and parks. The key factors influencing nest density and placement included proximity to trash bins, water sources, and tall trees, reflecting the magpie’s adaptability to urban environments and reliance on anthropogenic resources.

## 1. Introduction

Progressive urban development and the expansion of urbanised areas are common phenomena across Europe, including in Poland. These processes often lead to the homogenisation of bird species in urban environments, as certain species adapt to the changing conditions while others may struggle to thrive [1]. Since the second half of the 20th century, an increase in magpie numbers has been observed in urbanised areas within its range [2]. The size of magpie territories and the distances travelled to foraging sites depend on many factors, e.g., the availability of food resources, the environmental structure, and predator pressure. Studies have indicated that magpies exhibit smaller areal sizes in urban environments compared to those of rural populations [3]. This trend is clearly visible in Zielona Góra, Poland, where the urbanised area has grown significantly over the past two decades, from approximately 57 km^2^ to 278 km^2^ [4]. However, this expansion has not been accompanied by a proportionate increase in the city’s population, which has risen from around 120,000 to 138,932 inhabitants as of 2023 [4]. Recent studies have suggested that the magpie *Pica pica*, as an urban exploiter, has increasingly concentrated in the centre of large cities, leaving semi-open suburban areas in favour of highly urbanised environments [5,6]. Morelli et al. [7] explored how urbanisation filters bird species based on behavioural traits, such as nesting and feeding strategies, identifying the attributes that enable species like magpies to thrive in urban landscapes. Numerous studies have examined the effects of urbanisation on bird populations, including its impact on nest predation dynamics (offering insights into how urban environments reshape predator–prey interactions and affect species such as magpies) [8], avian survival in urban habitats (highlighting traits, such as omnivory and adaptability, both critical for successful urban colonisation by magpies) [9], and the role of urban bird communities (emphasising the adaptability of magpies and their ecological roles in urban ecosystems) [10]. Additionally, contemporary methodologies, employing citizen science, have been used to track the urban expansion of magpie populations, underlining their increasing prevalence in highly urbanised areas [11]. These studies have collectively provided a comprehensive perspective on how urban environments influence avian populations across Europe.

The present study aims to determine the current magpie population in Zielona Góra and to provide a detailed characterisation of the changes in nest site selection over the past 23 years, emphasising the transformation of urban habitats and their impact on magpie nesting behaviour. Studies on the urban bird populations in Zielona Góra have been conducted intensively over the past 40 years, with a particular focus on the magpie population [2,12,13,14,15]. The highest magpie densities were observed in residential areas developed during the late 1960s and early 1970s, where the density reached 39.9 pairs per km^2^.

The rapid increase in the Eurasian magpie population observed in the second half of the 20th century was evident in many European cities [2,16]. Among the factors responsible for this phenomenon were changes in urban green spaces, particularly the planting of fast-growing tree species [13]. Studies on the nesting ecology of magpies in various European cities have demonstrated a notable preference for poplar trees, *Populus* sp., as nesting sites (e.g., [12,14,17,18,19,20,21,22,23]).

Recently, many studies have highlighted a shift in tree species selection toward conifers [14,23,24,25,26]. In Poland, this trend correlates with an increase in the planting of coniferous trees, such as spruces, in suburban single-family housing estates as decorative greenery. This development, which has been prominent since the 1990s, has led to an increase in spruce trees being chosen by magpies for nesting. Spruces stand out in urban landscapes due to their size and provide highly attractive habitats, offering year-round protection for nests. Furthermore, recent publications have indicated that the magpie is concentrated in the centres of large cities, leaving the semi-open areas of the suburbs for highly urbanised areas [5,6].

The aim of this study is to report on the abundance and density of magpies in the different environments of the city. In addition, the selection of magpie nesting sites in Zielona Góra is characterised and the population dynamics of magpies are described based on the available literature. We focus also on urban habitat selection, with particular attention to the changes in magpie nesting preferences, including the tree species, habitat types, and microhabitats. Additionally, we aim to identify the specific environmental factors influencing nest placement and height, such as proximity to water sources, trash bins, building height, and surrounding vegetation. An additional objective is to determine the co-occurrence of small bird species in the immediate vicinity of the nests of given breeding pairs.

## 2. Materials and Methods

### 2.1. Field Study

Zielona Góra (51.9401° N, 15.5035° E) is a medium-sized city in western Poland with a population of 140,000. It is located in the Lubuskie province and covers an area of 278.3 km^2^. The urbanised area of Zielona Góra is surrounded by coniferous forests, and former agricultural land has been replaced by multi- and single-family housing developments. The following habitats were assumed to be urbanised areas: single-family housing estates, blocks of flats, industrial areas, allotments, old town, parks, and districts with a rural character. We excluded forested areas from the urbanised area.

**Figure 1:**
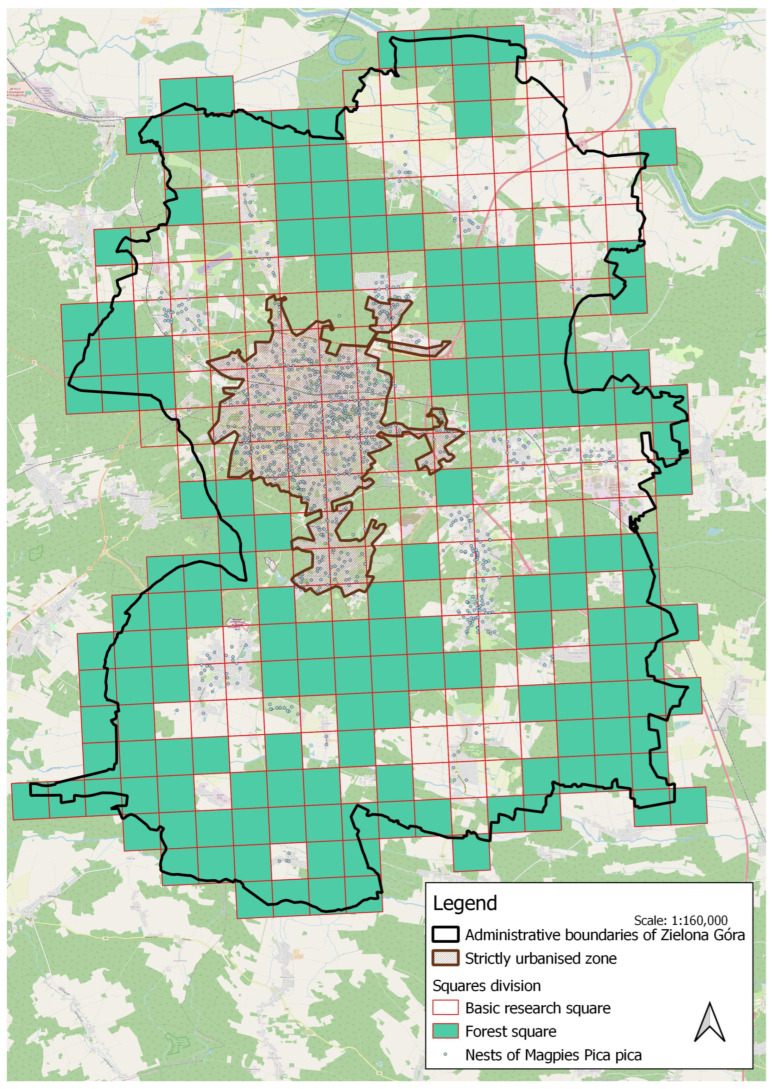
Study area, squares division, and land cover of the city of Zielona Góra. Each square is 1 km × 1 km. Basemap: © OpenStreetMap contributors, licensed under the Open Database License (ODbL).

In order to correctly compare data from previous publications, the magpie density survey in Zielona Góra was recalculated for two areas described below:

1. Current administrative boundaries of Zielona Góra without forests (only allotment gardens, blocks of flats, single-family housing estates, industrial zones, the old town, parks, and village districts with a rural character) has an area of 108.4 km^2^ (Figure 1).
2. Strictly urbanised zone (allotment gardens, blocks of flats, single-family housing estates, industrial zones, the old town, and parks) was established to compare data to the current year and includes the study boundaries proposed by Bocheński et al. [14], and has an area of 23.4 km^2^ (Figure 1).

The city has a temperate climate, with an annual mean temperature of 10.0 °C and 664 mm of precipitation. We divided Zielona Góra into 320 squares, each covering 1 km^2^ (Figure 1). Heavily forested squares that did not meet the habitat requirements of the studied species were excluded from the survey, leaving 162 plots for analysis. The division was made using QGIS software, where relevant polygons were delineated based on satellite images—a raster map with a scale of 1:1000 obtained from the Head Office of Geodesy and Cartography, Poland (Główny Urząd Geodezji i Kartografii, Polska).

### 2.2. Data Collection

The data were collected during two surveys in March and April 2022, to detect the highest number of present pairs once per season. Each nest was marked on a digital map using GPS devices. Within the designated squares, potential nesting sites for magpies were searched for, including rows of trees, bushes, individual trees, organised greenery, parks, and squares. Once a nest was located, observations focused on the breeding behaviour of the magpies, such as nest building, birds staying in the nest, and/or defending the territory. If no birds were observed near a nest, attention was given to the nest’s construction, e.g., compact structure and fresh materials. Experienced researchers paid special attention to indicators, such as fresh twigs in nests, adults carrying nesting materials, birds disturbed by human presence near nests, or adults staying in nesting areas or incubating.

During the control period the following data were collected: the tree species in which a nest was built, tree height, and height of nest position in the tree. Habitat types were also recorded, including allotment gardens, blocks of flats, single-family housing estates, industrial areas, old town, parks, and villages. Microhabitat types were categorised as single trees, tree clusters (2–3, 4–10, or more than 10 trees), and rows of trees. Within a 50 m radius of a nest, additional features, such as the distance to the nearest open water reservoir, the number of trash cans, and the mean number of building floors, were counted. Furthermore, within a 50 m radius of a nest, other bird species were recorded, focusing on their use of the area below the nest (e.g., foraging, singing males) to assess environmental quality [27]. Observations were conducted using binoculars. Tree height and nest placement height were measured using a Suunto altimeter PM5/1520 and visual estimation with an accuracy of 0.5 m. Spatial analysis was performed using QGIS v3.24 software.

### 2.3. Statistical Analysis

Exploration of avian species’ composition, regarding the main type of habitat, was performed by using the ‘heatmap’ function in R. It produces high-quality matrix showing the correlation between species and habitat, and runs clustering algorithm to visualise associations (between species and habitat and among species) with dendrograms. The heatmap was elaborated using the package ‘pheatmap’ for R [28].

Environmental features affecting the number of magpie nests in Zielona Góra and their height were tested by using generalised linear models (GLMs) [29]. A test of variance inflation factor (VIF) was applied to check for potential multi-collinearity among predictor variables, using the “car” package for R [30]. Only models with VIF < 6 were considered. The variable “tree cluster large” was removed because it produced a VIF > 9.

The first model was run accounting for variations in number of nests in each 1 km × 1 km square, regarding the tree height (mean), nearness to open water (nearest distance), trash cans (total number), building floors (mean), and percentage of microhabitat type (single tree, small tree clusters, medium tree clusters, tree rows, and other types). The second model was run accounting for variations in the mean nest height above the ground, regarding the number of nests, tree height, nearness to open water, trash cans, building floors, and percentage of microhabitat type (single tree, small tree clusters, medium tree clusters, tree rows, and other type). The models were fitted following a Poisson distribution for the number of nests, and normal distribution for the mean nest height, after determining the type of distribution for each response variable [31] using the package ‘MASS’ [32]. The selection of the best model was performed by ranking and weighting them according to their fit to the observed data (e.g., lower AIC value and higher weight, where the weight is the probability that the model is the best model given the candidate set included in the model selection procedure). The best-supported models were averaged to obtain parameter estimates. Model runs, selection, and averaging were performed using the ‘dredge’ function from the package ‘MuMin’ [33] and the package ‘lme4’ [34] for R.

All statistical tests were performed with R software version 4.1.1 [35].

## 3. Results

### 3.1. Magpie Population Density in Zielona Góra

A total of 953 magpie pairs and their occupied nests were recorded across the entire area of Zielona Góra. The average density across the current administrative boundaries of Zielona Góra, without forests, was 8.8 pairs/km^2^. In the strictly urbanised zones (allotment gardens, blocks of flats, single-family housing estates, industrial zones, the old town, and parks), the density was 27.7 pairs/km^2^.

The density of magpies varied significantly across the different habitat types in the city. The highest density was observed in the old town (1.2 km^2^), with 36.5 pairs/km^2^; followed by block-housing areas (7.8 km^2^), with 34.5 pairs/km^2^; parks (2.5 km^2^). with 19.7 pairs/km^2^; single-family housing areas (19.6 km^2^), with 18.5 pairs/km^2^; allotment gardens (3.8 km^2^), with 14.9 pairs/km^2^; industrial zones (9.9 km^2^), with 7.7 pairs/km^2^; and rural-type developments (10.9 km^2^), with 6.1 pairs/km^2^. In the forested and farmland areas (222.2 km^2^), the density was as low as 0.1 pairs/km^2^, while no magpies were recorded in cemeteries (0.4 km^2^) (0 pairs/km^2^). The number of nests across the different habitat types was statistically significant (Chi^2^ with Yates’ correction = 20.426; df = 6; *p* < 0.002).

### 3.2. Nest Distribution and Height Across Habitat Types

Magpie nests were found in 32 tree genera, as well as on an electricity pylon. The most frequently used tree species for nesting were *Picea* sp. (22.5%), *Populus* sp. (13.2%), *Acer* sp. (10.8%), *Quercus* sp. (10.6%), *Betula* sp. (9.3%), *Pinus* sp. (6.0%), and *Tilia* sp. (5.4%), with other species accounting for the remainder.

Table 1 presents the number of nests and the average nest height across the different habitat types in Zielona Góra. The overall average nest height across all habitat types was 11.8 m, with a standard deviation of 4.3 m. The highest number of nests was recorded in single-family housing estates (406 nests) and near blocks of flats (262 nests), while the lowest numbers were found in allotment gardens (47 nests) and in the old town (49 nests).

The highest average nest heights were observed in the old town (15.0 m) and parks (14.3 m), while the lowest average nest heights were recorded in allotment gardens (9.9 m), likely due to the lower tree heights in this habitat type. The greatest variation in nest height was found in villages (SD = 4.8) and in the old town (SD = 4.9). In contrast, the smallest variation in nest height was observed in allotment gardens (SD = 3.0).

### 3.3. Environmental Factors Influencing Nest Placement

**Figure 2:**
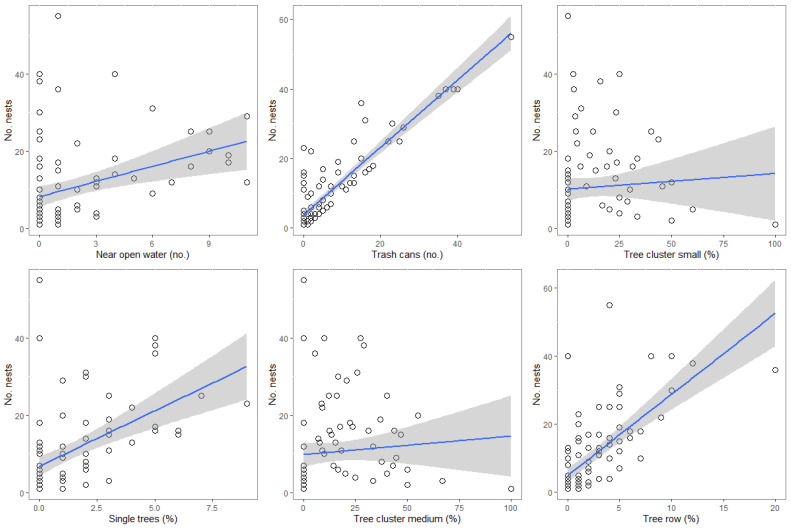
Associations between the number of magpie nests and the number of open water sources, trash cans, percentage of tree clusters of small and medium size, single trees, and percentage of tree rows in the city of Zielona Góra. Grey circle—observations of the number of nests depending on a given variable on the X axis; Grey areas—confidence intervals for the regression (95%); Blue line—regression line, overall trend of the relationship between the number of nests and the variable on the X axis.

Regarding the environmental features, magpie nests were more often located in areas with more sources of open water and trash cans, and with a high coverage of single trees, small- and medium-sized tree clusters, and tree rows (Table 2, Figure 2). The mean nest height was 11.8 m (SD: 4.3 m; max: 30 m; min: 3 m), whilst the mean tree height used for nesting was 13.3 m (SD: 4.9; max: 35 m; min: 3 m). The height of the nest location was positively associated with the overall height of the trees (Table 3), while it was negatively correlated with the average number of floors in the buildings surrounding the nest, and the percentage of medium-sized tree clusters (Table 3). The tables showing the selection of the best models are in the Supplementary Materials (Tables S1 and S2).

### 3.4. Co-Occurrence of Other Bird Species in Different Types of Habitats

**Figure 3:**
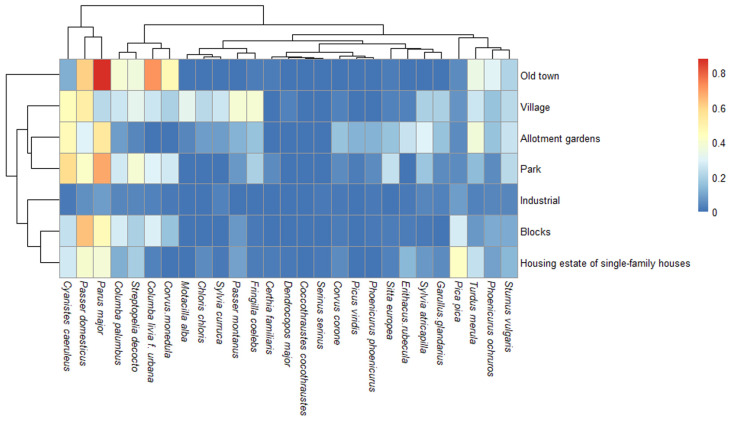
Heatmap indicating the association between the bird species and the type of habitat (e.g., old town, industrial, blocks of flats, etc.) and among the different bird species in the city of Zielona Góra. The correlation increases with the gradient of colours, from zero (dark blue) to near one (dark red).

The bird species with a relatively higher congruence regarding the use of habitats in the city of Zielona Góra (mainly occurring in the old town, parks, and the gardens of blocks of flats) were the great tit *Parus major*, feral pigeon *Columba livia f. urbana*, house sparrow *Passer domesticus*, and blue tit *Cyanistes caeruleus* (Figure 3).

## 4. Discussion

### 4.1. Magpie Population Density in Zielona Góra

The first data on magpie abundance in Zielona Góra date back approximately 100 years, when only seven pairs nested within the city limits [36]. Investigations have intensified since the 1980s, providing estimates of the population size and density during several periods. Between 1982 and 1997, the magpie density in the city increased from 5.9 pairs/km^2^ to 17.0 pairs/km^2^, with intermediate values of 10.4 pairs/km^2^ in 1987 and 13.7 pairs/km^2^ in 1992. Subsequent studies conducted in 2001 on selected urban areas in various environments reported an average density of 31.1 pairs/km^2^. In 2006–2007, their densities ranged from 26.9 to 27.6 pairs/km^2^ [14,15,37]. In the present study, conducted in 2022 across the entire expanded city area (boundaries adjusted in 2015), an average density of 27.7 pairs/km^2^ was recorded when recalculated for urban environments. Over the past 100 years, Zielona Góra experienced an initial rapid increase in the magpie population density, followed by stabilisation in recent years.

The range in magpie densities reported in this study across the various urban environments (from 7.7 pairs/km^2^ in industrial areas to 36.5 pairs/km^2^ in the old town) is comparable to or lower than previous findings for Zielona Góra. For example, in 2001, the densities ranged from 22.0 pairs/km^2^ in industrial zones to 39.9 pairs/km^2^ in block-housing areas [14].

In similar studies conducted in Gorzów Wielkopolski (also in western Poland), the maximum magpie density reached 22 pairs/km^2^, with an average density of 13.5 pairs/km^2^ in urbanised areas [26]. The densities observed in Zielona Góra are higher than those recorded in many other Polish cities, such as Siedlce (2.1 pairs/km^2^ [38]), Lublin (4.3 pairs/km^2^ [39]), and Szczecin (8.7 pairs/km^2^ [2]). However, they are comparable to those for Wrocław, where the densities range from 1.1 to 46 pairs/km^2^, depending on the habitat type [40,41,42].

Even the highest densities recorded in Zielona Góra (the old town and block-housing areas) are lower than those reported for block-housing estates in Poznań, where an average of 55 pairs/km^2^ was observed [43], and in Warsaw, where the densities reached 43 pairs/km^2^ and even exceeded 60 pairs/km^2^ [44,45]. Compared to Central European cities, the densities recorded in Zielona Góra are within the reported range of 17–57 pairs/km^2^ [2,46].

Comparing the magpie population sizes and densities across studies conducted in different habitat types and areas of varying sizes may distort the actual results [13]. This study, along with the cited works, highlights the substantial differences in magpie densities across urban habitat types. To a large extent, the density levels may depend on the structure of the urban greenery [13].

### 4.2. Nest Distribution and Environmental Factors Influencing Nest Placement

The analysis of magpie nest distribution based on environmental characteristics, taking into account the various types of urban habitats, reveals that magpies more frequently select locations near trash bins and open water. Furthermore, in urban habitats, magpies show a preference for nesting in single trees.

The analysis of magpie nest distribution based on environmental characteristics, taking into account the different urban habitat types, show that the number of magpie nests is higher in areas with a high coverage of single trees, medium clusters of trees, small clusters of trees, rows of trees, and more open water sources and trash cans. Magpie nests tend to be located higher in areas where the overall tree height is higher, but decrease in areas with more building floors and where the coverage of medium-sized tree clusters is higher.

Numerous studies have indicated that one of the factors contributing to high magpie densities in urban areas is the availability of human food waste [2,3,21,37,47,48,49,50,51]. Jerzak [37] suggested that the utilisation of anthropogenic food sources has been a critical factor in the success of urban magpie populations, highlighting this phenomenon as an example of urbanisation. In addition to population density increases, the role of anthropogenic food has also been noted in relation to the female body condition during breeding periods, the timing of breeding, and breeding success [52,53,54].

Pierotti and Annett [55] emphasised that anthropogenic food serves as an additional, rich resource available year-round in human-inhabited areas, primarily in the form of food scraps found in trash bins. Balanca [56] reported that waste-derived food constituted as much as 40% of the diet of chicks from nests located near buildings. In Zielona Góra, Jerzak [37] demonstrated a statistically significant positive correlation between the density of trash bins and magpie nests within the city. Similarly, Barszcz [57] found that magpies in Kraków more frequently built nests near trash bins.

While anthropogenic food waste may be suitable for adult magpies, it is less beneficial for chicks [58]. However, its role in the chick diet may increase with age or during periods of low availability of typical foods, such as during adverse weather conditions. This has been observed not only in magpies but also in other bird species [59,60,61,62,63,64]. Other studies have also documented magpies foraging in trash bins as a factor in their urban population growth [17,65,66,67].

Conversely, a detailed study on trash bin use as a food source conducted in Poznań (western Poland) found a low frequency of magpie visits to bins and no positive relationship between the number or quality of bins and magpie territory stability [68]. The author suggested that anthropogenic food from trash bins is not as critical as generally assumed. Considering that trash bins are only one of many sources of anthropogenic food, and that their availability and type vary over time, differ between cities, and may depend upon the degree of urbanisation, the significance of this food source likely varies among populations.

Our study also reveals the presence of open water as another factor influencing the number of local magpie nests. The availability of food and water are among the most important environmental factors limiting bird occurrence and abundance [69,70]. Food availability, in particular, can influence population size and structure [71]. Water is equally crucial for birds, not only for drinking but also for maintaining proper metabolism, thermoregulation, and plumage condition [72,73,74,75,76,77].

The primary food source for magpie nestlings in various habitats is invertebrates [78], and magpies primarily forage within their territories [47]. Considering the role of aquatic environments [79] and their positive impact on biodiversity in urban areas [80,81], including invertebrates, the presence of water within a magpie’s territory appears advantageous. A consistent water supply and increased food diversity near aquatic environments could positively affect the breeding success of magpies nesting in such areas.

Jerzak [37] identified individual trees, as well as tree alleys and clusters surrounded by grassy areas, as typical nesting sites for magpies in urban environments. Studies conducted in Zielona Góra in 2006–2007 showed that the highest number of nests were located in tree clusters, such as tree alleys [15]. In Gorzów Wlkp. in 2014, Ciebiera et al. [26] found that magpies most frequently selected tree alleys and individual trees. Similar results were obtained in Siedlce and Gdańsk, where trees planted in rows were most commonly used, followed by tree groups [38,82]. A slightly different result was observed in Białystok, where tree and shrub clumps were the most common nesting microhabitat (70.5%), while tree alleys were much less frequent [24]. Meissner and Żółkoś [22] suggested that magpies may prefer specific tree species rather than any particular spatial arrangement. Their study confirmed this by frequently identifying the Lombardy poplars *Populus nigra* ‘Italica’ as nesting trees, and noting a preference for trees growing individually or in pairs. A similar trend may currently exist in Zielona Góra, where spruces, which often grow singly or in small groups within urban greenery, are the most frequently chosen tree species. Meissner and Żółkoś [22] further suggested that the inconsistent results regarding magpie preferences for specific tree-grouping types, as presented in various studies (as cited above), may result from the fact that different species of trees have been planted in different spatial arrangements in each city.

The height at which magpie nests are located in urban areas may depend on pressure from humans and other mammals [37,44,83]. In some cities, it has also been found that nests located lower to the ground have a lower breeding success [84,85,86]. However, differences in nest height, both in urban and rural environments, may also result from differences in the species composition of the trees growing in these areas [38].

Over the span of 20 years, the average height of magpie nests in Zielona Góra decreased from 17.0 m to 11.8 m ([14], present study). This decrease is attributed to a change in the most commonly selected trees for nest building (formerly poplars, now spruces). Czechowski et al. [23] recorded a similar decline in nest height due to changes in tree species preference. Over the course of 17 years, magpies shifted their preference from deciduous to coniferous trees, which resulted in a decrease in nest height. Dense, inaccessible conifer trees, although shorter than the previously preferred deciduous species, provide safer nesting locations.

Our analyses show that the height of nest placement is proportional to the tree height and correlates with the average height of the surrounding buildings. This relationship between nest height and tree height has also been demonstrated in other cities [5,38]. It is believed that magpies, which begin breeding early, before trees shoot leaves, prefer taller trees to limit predators’ access to them [12,21,87]. This phenomenon has also been observed in other bird species [88]. The preference for nesting in tall trees, particularly poplars, has been characteristic of many cities in Poland [13,22]. The preference for poplars may be due not only to the high availability of this species in urban greenery and its height, but also to the structure of its canopy, which allows for nest placement at the top of the tree, limiting predator access [22]. Spruce, now the most commonly chosen tree in Zielona Góra, and poplar both offer the possibility (due to their canopy structure) of placing a nest at the very top.

More detailed studies are needed to explain the observed influence of the average height of buildings on nest height, but some explanations can be found from three aspects. First, the territorial behaviour of magpies, particularly their perching at the tops of trees, increases the visibility of their territory [89]. Buildings may act as a visual barrier, leading magpies to choose taller nesting trees, and consequently, higher nest placements. Kaczmarek [3] studied the effect of buildings acting as visual barriers and found that they could influence the reduction in distances between active magpie nests. Second, taller buildings lead to a higher population density in smaller areas, increasing human pressure and potential predator presence, such as that of cats. Third, such a relationship may also result from the presence (planting) and availability of tall trees, which are more common in areas with taller buildings than in single-family housing or historic city centres.

The current study confirms the recent trend of magpies building nests in coniferous trees, especially spruces, both in urban and suburban areas [5,23,24,26,44,48]. In the studies reviewed, 22.5% of nests were found in coniferous trees, while in 2001, only 2.9% of the nests in Zielona Góra were located in spruces. In Gorzów Wlkp. in 2014, the proportion of spruces among trees with nests was 20.1% [26]. In the South Bohemian region (Czech Republic), by the end of the second decade of the 21st century, 10.1% of magpie nests were found in coniferous trees, indicating that these changes have affected both urban and agricultural areas [25]. These findings suggest a decline in the dominance of poplars as the primary tree species for magpie nesting [22,37]. In Zielona Góra, over a period of 20 years, the proportion of poplars decreased from 38.7% to 13.2% ([14], present study). In the neighbouring city of Gorzów Wlkp., the proportion of spruces and poplars was similar (around 20%, with spruces predominating) [26]. The results of the present study indicate an increasing preference for coniferous trees, likely due to the growing popularity of such trees and their increased availability (fast-growing species). Several factors are cited as the primary causes for this shift: coniferous evergreen species (which do not shed leaves in winter), with their dense canopies, make nests less visible in early spring and thus less accessible to predators, as well as provide earlier opportunities for breeding [5,21,26]. A parallel can be drawn to the previous preference for poplars as nesting trees over the years, which has been described in earlier studies (tall trees with thin branches, which, despite the lack of leaves in early spring, helped reduce predator access). Changes in the choice and dominance of nesting trees may also indicate that magpies do not always select the most common tree species in cities, with this preference potentially being related to a city’s geographic location [2,5].

### 4.3. Co-Occurrence of Other Bird Species in Different Types of Habitats

The interactions between magpies and other specific bird species have been widely discussed in the literature [90,91], particularly between magpies and carrion crows, *Corvus cornix* [92,93]. These studies have examined the magpie’s impact on passerine species, demonstrating its influence on their breeding success, especially for open-nesting songbirds. However, many authors have emphasised that this phenomenon is influenced by various factors, and the effect of magpies on the population density of other species remains unclear [91]. In contrast, Morelli et al. [27] identified bird species, mostly territorial and non-migrant, that can serve as indicators of urban areas with high environmental quality (HEQ). Among the top ten indicator species, the blackbird *Turdus merula*, great tit, blue tit, house sparrow, and magpie were highlighted, with these species found in 33–47% of the monitored cities [27]. The authors also proposed a preliminary investigation into the relationship between HEQ species occurrences and magpie territories, defined as areas within 50 m of magpie nests in locations characterised by high green cover/diversity, low light pollution, and high avian community resilience. A study conducted in Zielona Góra revealed that, similar to other cities, the presence of great tits, feral pigeons, house sparrows, blue tits, and other birds associated with anthropogenic habitats was prevalent near magpie nests. These observations were not dependent on the height or type of tree, but appeared to be related to the availability of food resources, such as waste and other nearby vegetation and shrubbery. This further confirms the adaptability of magpies to urbanised environments, where food and suitable nesting spaces are more readily available. The presence of tree alleys and coniferous trees also enhances this inter-species association, providing diverse habitats for both magpies and the species they coexist with.

## 5. Conclusions

Over the past century, the Eurasian magpie population in Zielona Góra exhibited a rapid increase in density, followed by stabilisation in recent years. The magpie’s ability to adapt to urban environments, including its utilisation of anthropogenic resources and diverse habitats, has been a critical factor in its success. Changes in nesting preferences, including the shift from deciduous to coniferous trees (particularly spruces), highlight the species’ flexibility in response to urban greenery composition. These preferences reflect the advantages of dense canopies and reduced predation risk. The availability of anthropogenic food sources, proximity to water, and the structural diversity of urban greenery significantly influence nest distribution and density. While trash bins are a notable food source, their importance may vary among cities, reflecting differences in urbanisation and waste management. Magpie densities in Zielona Góra are higher than in many Polish cities and comparable to those in Central Europe. However, these densities remain lower than the highest recorded values in cities like Warsaw and Poznań, emphasising regional variations in urban habitat quality and resource availability. Decreasing nest heights over the past two decades correlate with a shift in the preferred tree species and urban building structures. Taller nesting trees near higher buildings mitigate human and predator pressures and enhance territory visibility. The presence of other urban bird species near magpie nests, such as great tits, feral pigeons, house sparrows, and blue tits, demonstrates the ecological role of magpies in maintaining urban biodiversity. These associations are influenced by shared habitat features and resource availability. This study underlines the importance of preserving diverse urban greenery, particularly coniferous trees and water sources, to support magpie populations and overall biodiversity. Urban planning should consider these elements to balance ecological needs with city development. Further studies are needed to explore the relationship between magpie nesting behaviour, tree species selection, and urban structural changes. Investigating the long-term trends in different cities will improve our understanding of urban bird ecology and inform conservation strategies.

## Figures and Tables

**Table 1:** Number and height (±standard deviation) of magpie nests in Zielona Góra for each investigated type of habitat.

Habitat Type	N	Nest Height (Mean)	Nest Height (SD)
Allotment gardens	47	9.9	3.0
Blocks	262	12.0	3.7
Housing estates with single-family houses	406	11.2	4.5
Industrial	80	11.8	4.2
Old town	49	15.0	4.9
Parks	47	14.3	4.1
Villages	62	11.5	4.8
Total	953	11.8	4.3

**Table 2:** Results of the best model accounting for the number of magpie nests in each habitat type in the city of Zielona Góra, regarding the environmental features.

Variable	Estimate	Standard Error	t Value	*p* Value
Intercept	1.186	0.092	12.87	*p* < 0.001
Open water (no.)	0.069	0.009	7.46	*p* < 0.001
Single tree (%)	0.007	0.002	3.708	*p* < 0.001
Trash cans (no.)	0.055	0.002	25.846	*p* < 0.001
Tree cluster, medium (%)	0.005	0.002	2.565	0.010
Tree cluster, small (%)	0.006	0.002	2.949	0.003
Tree row (%)	0.006	0.002	3.823	*p* < 0.001

**Table 3:** Results of the best model accounting for the magpie nest heights in each habitat type in the city of Zielona Góra, regarding the environmental features.

Variable	Estimate	Standard Error	t Value	*p* Value
Intercept	0.319	0.245	1.306	0.195
Building floors (mean)	−0.125	0.045	−2.786	0.007
Tree cluster, medium (%)	−0.008	0.003	−2.591	0.011
Tree height (mean)	0.892	0.018	50.065	*p* < 0.001

## Data Availability

The data supporting the findings of this study are available from the corresponding author upon reasonable request.

## Supplementary Materials

The following supporting information can be downloaded at: https://www.mdpi.com/article/10.3390/ani15050704/s1, Table S1: Model selection outputs, with the best model highlighted in bold. The full model accounts for the number of magpie nests in each habitat type in the city of Zielona Góra, regarding the environmental features. The models are ranked by the AICc values; Table S2: Model selection outputs, with the best model highlighted in bold. The full model accounts for the magpie nest height in each habitat type in the city of Zielona Góra, regarding the environmental features. The models are ranked by the AICc values.
