# Supplementary material for: Adaptation of Eurasian Magpie (Pica pica) to Urban Environments: Population Dynamics and Habitat Preferences in Zielona Góra (Poland) over 23 Years
Source: Animals (Basel). 2025 Feb 28;15(5):704. doi: 10.3390/ani15050704 (PMC11898477; doi:10.3390/ani15050704)
Supplement: Supplementary file 1 [file animals-15-00704-s001.zip › animals-3474824-supplementary.pdf]

Table S1. Model selection outputs, with the best model highlighted in bold. The full model accounts for the number of Magpie nests in each habitat type in the city of Zielona Góra, regarding environmental features. Models are ranked by AICc values.

(Int)	Building floors (mean)	Near open water	Other type land use	Single tree (%)	Trash cans	Tree cluster medium (%)	Tree cluster small (%)	Tree height (mean)	Tree row (%)	df	AICc	delta	weight
**1.186**		**0.06858**		**0.00684**	**0.05486**	**0.00497**	**0.00605**		**0.00619**	**7**	**622**	**0**	**0.381**
1.331		0.06910		0.00696	0.05571	0.00476	0.00638	-0.01217	0.00622	8	623.2	1.17	0.213
1.218	-0.02161	0.06701		0.00701	0.05611	0.00497	0.00612		0.00632	8	623.8	1.75	0.159
1.187		0.06883	-0.00028	0.00683	0.05487	0.00497	0.00604		0.00619	8	624.4	2.41	0.115
1.331	-0.01408	0.06797		0.00706	0.05640	0.00479	0.00637	-0.01044	0.00629	9	625.4	3.38	0.07
1.331		0.06935	-0.00029	0.00695	0.05573	0.00475	0.00637	-0.01217	0.00621	9	625.7	3.63	0.062

Table S2. Model selection outputs, with the best model highlighted in bold. The full model accounts for the Magpie nest height in each habitat type in the city of Zielona Góra, regarding environmental features. Models are ranked by AICc values.

(Int)	Building floors (mean)	Near open water	No. nests	Trash cans	Tree cluster medium (%)	Tree cluster small (%)	Tree height (mean)	df	AICc	delta	weight
**0.3194**	**-0.12460**				**-0.00820**		**0.89170**	**5**	**167.1**	**0**	**0.285**
0.2983	-0.12030	0.03001			-0.00915		0.88960	6	167.3	0.27	0.249
0.3296	-0.12850				-0.00839	-0.00284	0.89430	6	168.7	1.63	0.126
0.3158	-0.13530		0.00445		-0.00848		0.89030	6	168.7	1.68	0.123
0.3087	-0.12440	0.03082			-0.00938	-0.00306	0.89240	7	168.9	1.83	0.114
0.3276	-0.13410			0.00314	-0.00831		0.89100	6	169.1	2.06	0.102
